# Development of a valid and reliable pterygium surgery assessment scale for ophthalmology residents

**DOI:** 10.1186/s12909-021-02934-y

**Published:** 2021-09-27

**Authors:** Zhihua Zhang, Tianwei Qian, Bijun Zhu, Haiyun Liu, Xiaodong Sun, Xun Xu

**Affiliations:** 1 Department of Ophthalmology, Shanghai General Hospital, Shanghai Jiao Tong University, 100 Haining Road, Hongkou District, 200080 Shanghai, China; 2 National Clinical Research Center for Eye Diseases, Shanghai, China; 3 Shanghai Key Laboratory of Ocular Fundus Diseases, Shanghai, China; 4 Shanghai Engineering Center for Visual Science and Photomedicine, Shanghai, China; 5 Shanghai Engineering Center for Precise Diagnosis and Treatment of Eye Disease, Shanghai, China

**Keywords:** Pterygium surgery, Assessment scale, Reliability, Validity

## Abstract

**Background:**

Microsurgery training has become an important part of ophthalmology teaching and one of the main topics of examination. Accurate and effective evaluation of microsurgery skills is vital for the training and teaching of residents. In this study, we aimed to establish a pterygium surgery assessment scale for use by ophthalmic residents and evaluate its reliability and validity.

**Methods:**

Based on a literature search, experienced pterygium surgeons developed the preliminary scale according to the standard surgical procedure. The preliminary scale and a questionnaire were sent to teaching and research experts in the field for feedback. Face and content validity and reliability of the scale were determined by rounds of modifications based on expert feedback. For construct validity, existing assessment scales were obtained and a range of factors were tested.

**Results:**

Nineteen expert surgeons completed the questionnaire and modifications were made until all surgeons agreed on the final scale. Good construct validity was found by evaluation against 257 existing scales. For reliability, 280 evaluation scales were completed. Inter- and intra-rater reliability analysis both found Intraclass Correlation Coefficient (ICC) > 0.8 for all items and total scores.

**Conclusion:**

The pterygium surgery assessment scale developed in this study has good reliability and validity, and is an effective measurement tool for the evaluation of ophthalmology residents’ pterygium surgical skills.

In 2002, the American Board of Ophthalmology added surgery and surgical skills as the seventh independent category to the six categories previously defined by the Accreditation Council for Graduate Medical Education (ACGME) for evaluating competency as an educational outcome of residency programs [1]. Over time, microsurgery training has gradually become an important part of ophthalmology teaching and one of the main topics of examination for ophthalmology residency programs worldwide [2, 3]. At the beginning, the evaluation of surgical skills has been based on the subjective impression of examiners with a lack of standardized evaluation, limiting the consistency and credibility of the assessment. Thus, the International Council of Ophthalmology (ICO) has established several standardized assessment scales related to cataract, strabismus, ptosis and other ophthalmic surgery, with excellent feedback and application effect [4–9]. After integrating with the ICO, ophthalmologists in China began to use these scales. However, they mainly focus on surgery conducted by high-level staff and not by ophthalmic residents. Therefore, for the training and teaching of surgical skills, evaluation methods for appropriate levels of surgery are needed.

Pterygium is a common ocular surface disease, the standard surgical treatment of which is pterygium excision and conjunctival autograft transplantation [10]. The procedure involves the use of instrumentation and suturing under a microscope, offering opportunities to fully assess basic microsurgical skills. Therefore, in Shanghai, pterygium surgery is mandated as a periodical exam in the residency program. Accurate and effective evaluation of this procedure is vital for the training and teaching of residents [11, 12], but no appropriate standardized evaluation scale exists in China. We previously developed an efficient and reliable scale for the assessment of corneal suture technique [13]. In the present study, we aim to establish a similar scale for pterygium surgery to evaluate the surgical competency of residents and improve traditional ophthalmic surgery teaching methods.

## Methods and materials

### Development of the assessment scale

**Fig. 1:**
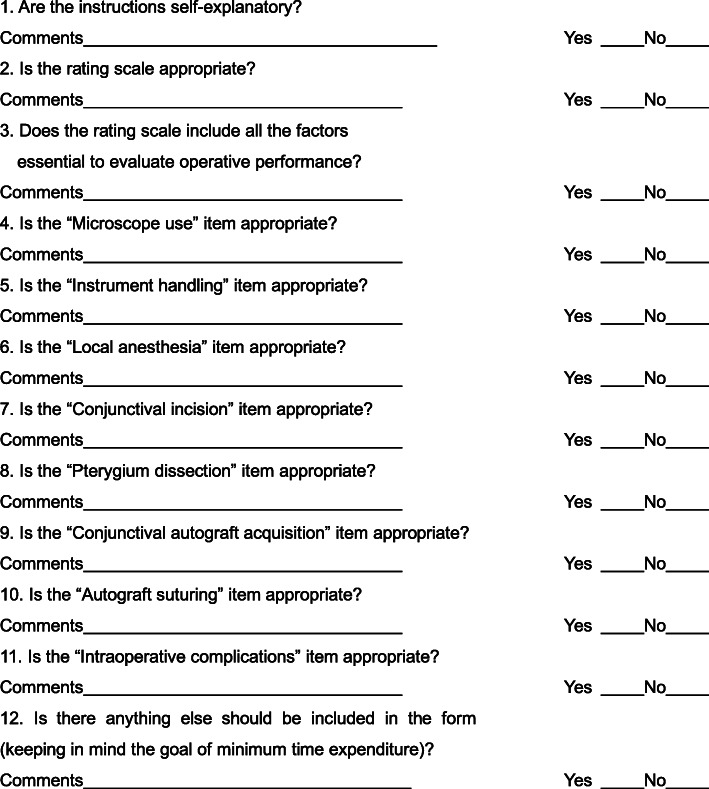
Questionnaire sent to experts to determine the face and content validity of the assessment scale

Two surgeons highly experienced in pterygium surgery developed the scale on the basis of a literature search and knowledge of the standard surgical procedure. Scale design took into consideration the examination syllabus with the aim of ensuring that the scale could assess basic surgical skills, pterygium dissection and ocular surface reconstruction. Any discrepancies between the two surgeons were discussed with a third person to develop the preliminary scale. This was sent with the feedback form (Fig. 1) to experts from several teaching and research offices including one member of the committee of Shanghai standardized residency program. Before completing the feedback form, the purpose and significance of the research were clearly explained to them and instructions for completion were provided. The experts were asked to read the scale carefully and then complete the questionnaire. They were also asked to identify any difficult or ambiguous questions in the scale and whether the wording of each item was clear.

### Surgery recording

All pterygium surgeries were performed in theatre by pterygium excision and conjunctival autograft transplantation and all necessary instruments were placed on the sterile operating platform. For each resident, the entire procedure was recorded and stored using a high-definition video system. Videos of residents from the first, second and third years of the residency program were randomly selected using a stratified sampling method. Eight such videos recorded pterygium surgery were chosen. These videos were conducted by two first year, three second year and three third year residents.

### Reliability and repeatability of the assessment scale

Twenty senior surgeons from different specialties formed an expert evaluation team, each independently viewing the eight videos and completing the evaluation scale. The experts were masked to residents’ rotation level during the evaluation. Three months later, each expert was asked to repeat this process, viewing each video and completing the scale for each. To minimize recollection of the first evaluation, the order in which videos were viewed differed between the two occasions. Intraclass correlation coefficient (ICC) was used to test interrater reliability and intra-rater repeatability [14, 15]. ICC ranges from 0 to 1, with higher values indicating better reliability. ICC values greater than 0.7 were considered to indicate reliability [16, 17]. SPSS 23.0 software (IBM) was used for statistical analysis, and *P* < 0.05 was considered statistically significant.

### Construct validity of the assessment scale

After reliability testing, the scale was applied in examination of residents conducting pterygium surgery. The 12 items in the scale were organized into three categories including basic surgical skills (five items: preoperative preparation, local anesthesia, microscope use, instrument handling, and postoperative clean up); pterygium dissection (four items: conjunctival incision, pterygium head dissection, pterygium body separation and dissection and remove of subconjunctival tissue; and ocular surface reconstruction (three items: hemostasis, conjunctival autograft acquisition, and autograft suturing). All 12 items were scored independently. Amos software (version 24; SPSS, Inc., Chicago, IL, USA) was used and the values of Chi squared (χ^2^), degrees of freedom (df), goodness of fit index (GFI), adjusted goodness of fit index (AGFI), average variance extracted (AVE), composite reliability (CR), and root mean square error of approximation (RMSEA) were calculated to evaluate the construct validity of the scale [18, 19].

## Results

### Refinement of the assessment scale

Nineteen experts completed the questionnaire. Five experts suggested including the assessment of “preoperative preparation and post-operative management” because these skills are included in the video, and are part of the surgical procedure. Three experts suggested modified wording more conducive to understanding. Seven experts suggested using three independent items including “pterygium head dissection”, “pterygium body part dissection” and “removal of residual pterygium” to replace “pterygium dissection”, for detailed evaluation of surgery. Two experts suggested adding “hemostasis” to the scale, because it is an important skill in pterygium surgery and can affect prognosis. Five experts suggested removing the assessment of “intraoperative complications” from the scale due to likely inaccuracies in assessment. Each expert’s opinions and suggestions were fully considered, and those deemed appropriate were included in the evaluation scale. Further rounds of feedback and modification were made until all surgeons agreed on the final scale. In this way, face and content validity were achieved from these surgeons’ suggestions [3].

The final assessment scale is shown in Table 1. The scale included five basic surgical skills (preoperative preparation, microscope use, instrument handling, local anesthesia and postoperative management) and seven pterygium surgical skills (conjunctival incision, pterygium head dissection, pterygium body part separation, removal of residual pterygium, hemostasis, conjunctival autograft acquisition, suturing). Each item was rated on a 5-point Likert scale, with each point anchored by behavioral descriptors.

**Table 1:** Assessment Scale for Pterygium Surgery

DATE RESIDENT EVALUATOR	1	2	3	4	5	Score
Preoperative preparation	Failed to wear hat, mask and gloves	Failed to wear two of the three	Failed to wear one of the three	Wearing hat, mask and gloves correctly	Wearing hat, mask and gloves smoothly	
Microscope use	Off-center and refocusing constantly	Off-center and refocusing frequently	Out of center and focus occasionally	Stay in center and focus constantly	Fluid moves with microscope	
Instrument handling	Constantly makes tentative and awkward moves with inappropriate use of instruments	Frequently makes tentative and awkward moves with instruments	Fair use of instruments but occasionally stiff or awkward	Competent use of instruments	Fluid moves with instruments	
Local anesthesia	Unable to complete; does not understand the injection site and dose of lidocaine	Barely completed; the dose and site were not appropriate	Almost completed; the dose and site were almost appropriate	Completed; the dose and site were appropriate	Smoothly completed; injection of anesthetics under bulbar conjunctiva of pterygium body with accurate dose	
Conjunctival incision	Failed to make the incision	Barely made the incision; the location and length were not appropriate	Almost completed; the location and length were almost appropriate	Completed; the location and length were appropriate	Smoothly completed; incision was made 1 mm outside and parallel to the limbus. The length of the incision was 0.5 mm across the upper and lower edges of pterygium	
Pterygium head dissection	Failed to dissect	Less than 60 % of pterygium head was dissected from cornea	60 to 80 % of pterygium head was dissected from cornea	More than 80 % of pterygium head was dissected from cornea	Fully dissected pterygium head from cornea in its anatomical plane	
Pterygium body separation and dissection	Failed to separate pterygium body from conjunctiva and sclera	Less than 60 % of pterygium body was separated and dissected from conjunctiva	60 to 80 % of pterygium body was separated and dissected from conjunctiva	More than 80 % of pterygium body was separated and dissected from conjunctiva	Fully dissected pterygium body from conjunctiva without damaging episcleral vessels	
Removal of subconjunctival tissue	Failed to remove	Less than 60 % of subconjunctival tissue was removed	60 to 80 % of subconjunctival tissue was removed	More than 80 % of subconjunctival tissue was removed	Full removal of fibrovascular tissue leaving a thin and smooth conjunctival rim	
Hemostasis	Failed to complete hemostasis; the operation field is unclear	Barely completed, the hemostasis was not timely and effective	Almost completed, the hemostasis was almost timely and effective	Completed, the hemostasis is timely and effective	Perfectly completed hemostasis with minimal use of cautery	
Conjunctival autograft acquisition	Failed to obtain	The size and location of the conjunctival autograft was not appropriate; irregular cut in borders or buttonhole of the graft	The size and location of the conjunctival autograft was almost appropriate; some residual Tenon’s on the graft	The size and location of the conjunctival autograft was appropriate; few residual Tenon’s on the graft	Perfect size and location of the conjunctival autograft; thin and even graft	
Autograft suturing	Failed to suture, or sutured the graft upside and down	Sutures are completed with difficulty; the graft is not well secured by sutures	Sutures are completed with little difficulty; the graft is almost secured by sutures	Sutures are completed properly; the graft is secured by sutures	Smooth and perfect suturing with good space and tension	
Postoperative clean up	Failed to clean the table. Failed to settle the microscope and instruments. Failed to remove hat, mask and gloves properly	Failed to do two of the three things	Failed to do one of the three things	Completed all of the three things	Disposed of medical waste in correct bins. Settled the microscope and instruments. Removed the hat, mask and gloves correctly	

If the description of a score cannot be satisfied, a lower grade of score will be allotted

### Reliability and repeatability of the assessment scale

The 20 expert surgeons who viewed the videos and completed evaluation for each had specialties in cataract (4), glaucoma (2), cornea (4), strabismus (2) and retinal disease (8). Fifteen of the 20 repeated this process 3 months later. A total of 280 evaluation scales (160 on the first occasion and 120 on the second) were completed. All experts said that they were able to complete the scale within a 5-min period.

The interrater reliability of each item and overall score, including all 20 evaluators involved on the first occasion, are shown in Table 2. All ICC values of all items including the total score were greater than 0.8 (0.852–0.992), and 69 % of the data were greater than 0.9. The “local anesthesia” item showed the highest interrater reliability (0.992, 95 % CI 0.982–0.998).

**Table 2:** Interrater reliability of twenty observers for pterygium surgery assessing scale

	ICC	95% CI	
		Lower bound	Upper bound
Preoperative preparation	0.871^***^	0.693	0.969
Microscope use	0.992^***^	0.982	0.998
Instrument handling	0.895^***^	0.750	0.975
Local anesthesia	0.852^***^	0.647	0.965
Conjunctival incision	0.908^***^	0.780	0.978
Pterygium head dissection	0.973^***^	0.850	0.985
Pterygium body separation and dissection	0.933^***^	0.841	0.984
Removal of subconjunctival tissue	0.904^***^	0.771	0.971
Hemostasis	0.976^***^	0.944	0.994
Conjunctival autograft acquisition	0.863^***^	0.673	0.967
Autograft suturing	0.914^***^	0.795	0.980
Postoperative clean up	0.924^***^	0.819	0.982

*ICC* intraclass correlation coefficient, *CI* confidential interval

^***^P < 0.001

Table 3 shows intra-rater reliability (repeatability) of each evaluator. The ICC values of all items and total score were greater than 0.8, and 62 % of the data were greater than 0.9, the item “conjunctival autograft acquisition” showing the highest repeatability (0.962, 95 % confidence interval 0.945–0.974).

**Table 3:** Intrarater reliability (repeatability) for pterygium surgery assessing scale

Item	ICC	95% CI	
		Lower bound	Upper bound
Preoperative preparation	0.916^***^	0.879	0.941
Microscope use	0.923^***^	0.890	0.947
Instrument handling	0.883^***^	0.832	0.919
Local anesthesia	0.846^***^	0.779	0.893
Conjunctival incision	0.894^***^	0.847	0.926
Pterygium head dissection	0.914^***^	0.876	0.940
Pterygium body separation and dissection	0.934^***^	0.905	0.954
Removal of subconjunctival tissue	0.841^***^	0.772	0.889
Hemostasis	0.962^***^	0.945	0.974
Conjunctival autograft acquisition	0.909^***^	0.869	0.936
Autograft suturing	0.837^***^	0.767	0.887
Postoperative clean up	0.908^***^	0.867	0.936

*ICC* intraclass correlation coefficient, *CI* confidential interval

^***^P < 0.001

### Validity of the assessment scale

Construct validity of 257 assessment scales was analyzed (Table 4). In this classification model, the χ^2^/df = 2.699 < 3, goodness of fit index (GFI) = 0.931 > 0.9 and adjusted goodness of fit index (AGFI) = 0.902 > 0.9, which means that model fit is fair. Average variance extracted (AVE) was used to reflect convergent validity. The AVE values of the three categories (basic surgical skills, pterygium dissection and ocular surface reconstruction) were 0.584, 0.571 and 0.631. CR values were 0.874, 0.842 and 0.835, and the RMSEA value was 0.043 < 0.05. These results showed good construct validity.

**Table 4:** The construct validity of the scale

			Estimate	S.E.	*P*	AVE	CR
Preoperative preparation	<---	Basic surgical skills	0.640			0.584	0.874
Local anesthesia	<---	Basic surgical skills	0.753	0.118	< 0.001		
Microscope use	<---	Basic surgical skills	0.731	0.107	< 0.001		
Instrument handling	<---	Basic surgical skills	0.762	0.106	< 0.001		
Postoperative clean up	<---	Basic surgical skills	0.911	0.116	< 0.001		
Conjunctival incision	<---	Petrygium dissection	0.739			0.571	0.842
Pterygium head dissection	<---	Petrygium dissection	0.753	0.099	< 0.001		
Pterygium body separation and dissection	<---	Petrygium dissection	0.768	0.086	< 0.001		
Removal of residual pterygium	<---	Petrygium dissection	0.763	0.094	< 0.001		
Hemostasis	<---	Ocular surface reconstruction	0.781			0.631	0.835
Conjunctival autograft acquisition	<---	Ocular surface reconstruction	0.682	0.074	< 0.001		
Autograft suturing	<---	Ocular surface reconstruction	0.904	0.098	< 0.001		

*S.E.* Standard error, *AVE* Average variance extracted, *CR* Composite reliability

## Discussion

At the beginning of the 21st century, ophthalmology training in China remains nonsystematic, and the quality of training varies between hospitals. In an effort to address this situation, Shanghai established standardized training programs for junior residents (in 2010) and senior residents (in 2014). Those programs play an extremely important role in ensuring the professional standard of ophthalmologists and the quality of indispensable medical services. The ophthalmologist Training Committee of Shanghai standardizes these programs into 3 years of training, the final year of which includes evaluation of surgical skills. In Shanghai, assessment of junior residents’ surgical skill is based on their ability to suture corneal ruptures on pig eyes, and senior residents’ skills are assessed by performance of pterygium excision and conjunctival autograft transplantation in theatre. We have previously developed an assessment scale for the process of suturing corneal rupture, and its validity and reliability have been confirmed in practice [13]. However, the assessment of pterygium surgery remains subjective and is prone to factors such as unconscious bias [12, 20]. Therefore, a critical need exists for a valid and reliable assessment tool.

In this study, we designed an evaluation scale for pterygium surgery conducted in China. The principles of the design are: (1) feasibility (rapid and easy to use); (2) whole-procedure evaluation; (3) surgical skill assessment at different rotation levels; (4) feedback and summative evaluation to improve skills and competencies. The final scale consists of 12 items, including five on basic surgical techniques and seven on pterygium surgery. The scale uses a 5-point Likert scoring system, and each score has a detailed score description. Zarei-Ghanavati et al. [3] also developed an assessment rubric for pterygium surgery. Our scale is similar to theirs in structure, but different in content. For example, we included the evaluation of basic surgical skills such as microscope use and instrument handling since they are important aspects of microsurgery. However, items beyond resident level such as Mitomycin-C application and fibrin glue usage are not included. In the scale, percentage score categories such as 60 %, 80 % were used, for more accurate and objective evaluation of the scale. Moreover, the scale is relatively simple and all evaluators reported completion within a period of 5 min, suggesting that it can be applied in rapid and large-scale resident assessment. More importantly, Zarei-Ghanavati et al. did not test the construct validity and repeatability level of their scale. In this study, the scale was completed 537 times (280 for reliability and 257 for validity), and validity as well as reliability were demonstrated. For validity, a level of face and content validity was established by considering all comments and incorporating appropriate suggestions into the assessment scale. Good construct validity found using GFI, AGFI, AVE, CR and RMSEA. We used Intraclass correlation coefficient (ICC) to test interrater reliability and intra-rater reliability. Although ICC is something of a blunt tool, any disagreement greater than 1 point among teachers would represent a major problem. Fortunately, this problem only occurred twice, when one same teacher evaluated one same student. The two assessment items were “instrument handling” and “local anesthesia”. The scores given by the teacher was two points worse than those given by some other teachers. The student is a first-year resident without much surgical experience, and the teacher is a very strict supervisor. She was more rigorous than other teachers in score evaluation, which might lead to the score gap. Nevertheless, all ICC values of all items were more than 0.8. Note that ICC values greater than or equal to 0.75 indicate high reliability [17, 21].

In this study, we developed a comprehensive and widely applicable assessment scale to assess the key components of pterygium surgery. The scale will provide a practical and standardized scoring method for resident examination, and the pass mark of each item can be set as > 3 points. It uses an analytical scoring system, including observable and measurable components of surgery. This will help educators to reduce the subjectivity of evaluation, record any weaknesses and give appropriate, individualized feedback based on the assessment scale. It is hoped that this tool will provide a structured template for other programs to evaluate residents’ surgical skills.

## Data Availability

The datasets used and/or analysed during the current study available from the corresponding author on reasonable request.

## Abbreviations

ICO: International Council of Ophthalmology
ICC: Intraclass correlation coefficient
χ^2^: Chi squared
df: Degrees of freedom
GFI: Goodness of fit index
AGFI: Adjusted goodness of fit index
AVE: Average variance extracted
CR: Composite reliability
RMSEA: Root mean square error of approximation
